# Colonic Fermentation Promotes Decompression sickness in Rats

**DOI:** 10.1038/srep20379

**Published:** 2016-02-08

**Authors:** Sébastien de Maistre, Nicolas Vallée, Emmanuel Gempp, Kate Lambrechts, Pierre Louge, Claude Duchamp, Jean-Eric Blatteau

**Affiliations:** 1 Service de Médecine Hyperbare et Expertise Plongée, HIA Sainte-Anne, BP600, Toulon Cedex 9, France; 2 Équipe Résidente de Recherche Subaquatique Opérationnelle, Institut de Recherche Biomédicale des Armées, BP 600, Toulon Cedex 9, France; 3 LEHNA, UMR 5023-CNRS/UCBL, Université Claude Bernard Lyon 1, 43 Bd du 11 Novembre 1918, Villeurbanne Cedex, France

## Abstract

Massive bubble formation after diving can lead to decompression sickness (DCS). During dives with hydrogen as a diluent for oxygen, decreasing the body’s H2 burden by inoculating hydrogen-metabolizing microbes into the gut reduces the risk of DCS. So we set out to investigate if colonic fermentation leading to endogenous hydrogen production promotes DCS in fasting rats. Four hours before an experimental dive, 93 fasting rats were force-fed, half of them with mannitol and the other half with water. Exhaled hydrogen was measured before and after force-feeding. Following the hyperbaric exposure, we looked for signs of DCS. A higher incidence of DCS was found in rats force-fed with mannitol than in those force-fed with water (80%, [95%CI 56, 94] versus 40%, [95%CI 19, 64], p < 0.01). In rats force-fed with mannitol, metronidazole pretreatment reduced the incidence of DCS (33%, [95%CI 15, 57], p = 0.005) at the same time as it inhibited colonic fermentation (14 ± 35 ppm versus 118 ± 90 ppm, p = 0.0001). Pre-diveingestion of mannitol increased the incidence of DCS in fasting rats when colonic fermentation peaked during the decompression phase. More generally, colonic fermentation in rats on a normal diet could promote DCS through endogenous hydrogen production.

Scuba (self contained underwater breathing apparatus) diving is an increasingly popular activity, especially among older individuals who may be unfit and even under medication. Scuba diving can lead to bubble formation in peripheral tissues as a result of dissolved gas changing phase during decompression. When too many bubbles are generated in bloodstream and tissues, signs and symptoms referred to decompression sickness (DCS) can occur^1^. It is generally accepted that these bubbles form from pre-existing gaseous nuclei on vessel walls^2^ and that the amount of venous gas emboli is linked to the risk of DCS^3^,^4^. Bubbles in the blood activate the vascular endothelium, stimulate pro thrombotic phenomena and induce inflammation; platelet and leukocyte activation have been observed together with increases in the production of cytokines and cell adhesion factors^5^,^6^. It is now accepted that severe DCS is a systemic pathophysiological process that can induce reactions leading to ischemic damage in the spinal cord and brain^7^. Neurological damage in the spinal cord and brain underlies the most serious symptoms of DCS^6^. Even after standard treatment with hyperbaric oxygen, 20–30% of the divers suffering from neurological DCS do not fully recover at the time of discharge^8^. Identifying and managing factors that underlie desaturation accidents is therefore a major challenge.

It has been suggested that the gut microbiota might influence the occurrence of DCS^9^,^10^,^11^. Hydrogen-metabolizing microbes (the human colonic archae *Methanobrevibacter smithii*) were introduced into the large intestines of pigs or rats. *M. smithii* converts H_2_ and CO_2_ to methane and water. The goal was to remove some of the hydrogen taken up by the body during hyperbaric exposure in the course of dives with H_2_ as the diluent to O_2_ in the breathing gas mixture. Removing approximately 5–10% of the body burden of H_2_ reduced the risk of DCS by approximately 50%. Moreover, the higher the activity of the microbes introduced was, the lower the risk of DCS was (logistic regression, p < 0.05)^10^. Supplemental washout of the diluent gas as a result of microbial metabolism inversely correlated with DCS risk in a dose-dependent manner^12^. Furthermore, H_2_ metabolism by the native intestinal microbiota of pigs may protect against DCS following a H_2_ simulated dive^13^.

Conversely, bacterial fermentation of undigested sugars in the large intestine generates hydrogen, some of which diffuses around the body^14^. This endogenous hydrogen could exacerbate DCS even during dives with another gas than hydrogen as the diluent. It could directly increase inert gas load during hyperbaric exposure and contribute to bubble formation during the decompression phase.

The aim of this study was to assess whether stimulating colonic fermentation in fasting rats by feeding them mannitol (MNL) before the dive would increase the incidence of DCS. Inhibiting this fermentation pathway by administering oral metronidazole (MTZ) was also tested. After a rapid decompression, DCS signs and cell counts (platelets, leukocytes) were compared in rats force-fed with MNL and controls, either conditioned with MTZ or not.

## Results

### Weight

Weights were similar six days before compression in both groups (384 ± 36.5 g with MTZ versus 406 ± 37 g; NS).

Weight loss between force-feeding time and removal from the hyperbaric chamber was no different whether rats were force-fed with MNL or water (-0.5 ± 2.2% versus -0.6 ± 2.2%; NS).

### Hydrogen and carbon dioxide in exhaled air

**Figure 1:**
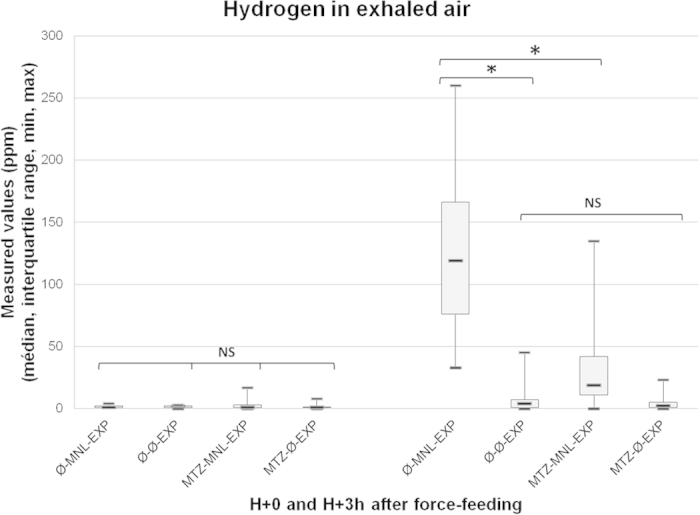
Hydrogen in exhaled air just before and 3 hours after force-feeding in rats. *denotes p < 0.05 between the groups.

In the group without MTZ, more hydrogen was exhaled one hour before compression by rats force-fed with MNL than those force-fed with water (118 ± 90 ppm versus 3 ± 6 ppm; p = 0.0001). In MNL-fed rats, the increase was significantly lower among those treated with MTZ (14 ± 35 ppm versus 118 ± 90 ppm; p = 0.0001). In water-fed rats, MTZ had no effect on hydrogen exhalation (Fig. 1).

In the group without MTZ, carbon dioxide production was the same one hour before compression in rats force-fed with MNL as in those force-fed with water (9.9 ± 4.1 ml.min^−1^ versus 8.8 ± 2.5 ml.min^−1^; NS). In MNL-fed rats, MTZ intake was not accompanied by a significant change in carbon dioxide production (9.9 ± 5.0 ml.min^−1^ versus 9.9 ± 4.1 ml.min^−1^; NS).

### Behavior and clinical observations

**Figure 2:**
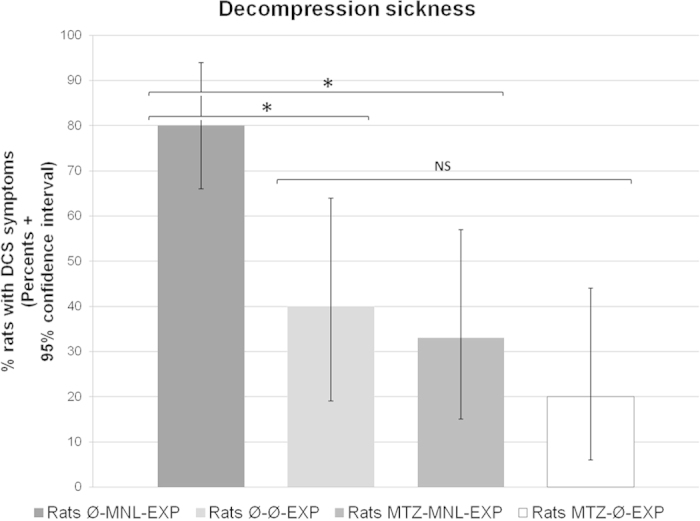
Percents of symptomatic rats suffering from decompression sickness (DCS) within 30 min after surfacing. *denotes p < 0.05 between the groups.

In the group without MTZ, the incidence of DCS of all types (death, neurological signs, labored breathing) was significantly higher in rats force-fed with MNL than in those force-fed with water (80% ± [56,94] versus 40% ± [19,64]; p = 0.0098) (Fig. 2). Death rates were not significantly different (65% ± [41,85] versus 35% ± [15,59]; p = 0.0578). In surviving rats, the incidence of neurological symptoms was significantly higher in MNL-fed rats (43% ± [10,82] versus 0% ± [0,25]; p = 0.0307).

In MNL-fed rats, MTZ intake significantly reduced the incidence of DCS (33% ± [15,57] versus 80% ± [56,94]; p = 0.0026) (Fig. 2) and the death rate was significantly lower (29% ± [11,52] versus 65% ± [41,85]; p = 0.0194). The incidence of neurological symptoms in surviving rats was not significantly different (7% ± [0,32] versus 43% ± [10,82]; p = 0.0766).

In water-fed rats, MTZ had no influence on the incidence of DCS (20% ± [6,44] versus 40% ± [19,64]; NS) (Fig. 2), deaths (15% ± [3,38] versus 35% ± [15,59]; NS) or neurological signs.

Finally, no deaths, neurologic signs or labored breathing were found in the sham pressurization group.

### Blood cell counts

In the sham pressurization group, there was no significant variation from force-feeding at the time of removal from the chamber, and no variation difference in hematological parameters between rats force-fed with MNL and those force-fed with water: platelets (-4.8 ± 18.2% versus -3.8 ± 16.5%; NS), white blood cells (-6.9 ± 37.4% versus +5.1 ± 22.0%; NS) or hematocrit (-1.9 ± 21.7% versus +9.2 ± 30.6%; NS).

**Figure 3:**
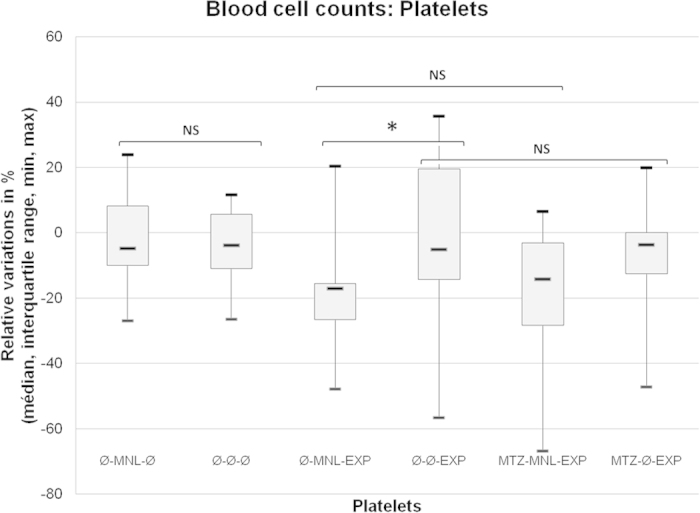
Percents of platelets consumption after decompression from the baseline. *denotes p < 0.05 between the groups.

In the group without MTZ, a significantly greater decrease was observed in platelet count at the time of removal from the chamber in rats force-fed with MNL compared to those force-fed with water (-17.1 ± 11.0% versus -5.0 ± 33.7%; p = 0.0358) (Fig. 3). Prior MTZ intake did not affect the platelet count in either rats force-fed with MNL (-14.1 ± 25.2% versus -17.1 ± 11.0%; NS) or in rats force-fed with water (-3.6 ± 12.6% versus -5.0 ± 33.7%; NS).

The white blood cell count was slightly lower at the time of removal from the chamber in rats force-fed with MNL with no prior MTZ intake (-40.0 ± 30.0%; p  =  0.0781), which was not seen in either rats force-fed with water (3.8 ± 65.4%; NS) or those force-fed with MNL after MTZ treatment (-6.3 ± 51.3%; NS). However, variation differences between these subgroups were not significant.

Hematocrit at force-feeding time was not different between groups with and without MTZ (44.6 ± 6.6% versus 47.3 ± 7.4%; NS). Hematocrit variation between force-feeding time and the time of removal from the chamber was not different between rats force-fed with MNL and those force-fed with water (-0.4% ± 30 versus 21 ± 1.6. 1%; NS).

### Arterial blood gases

In the group without MTZ, there was no significant difference in pH between rats force-fed with MNL and those force-fed with water (7.38 ± 0.05 versus 7.37 ± 0.07; NS) or in partial carbon dioxide pressure (44.1 ± 6.5 mmHg versus 45.8 ± 7.4 mmHg; NS).

## Discussion

We have shown in rats that ingesting MNL four hours before a dive—so that peak hydrogen production coincides with decompression—increases the incidence of DCS according to both clinical criteria (death, neurological signs, labored breathing) and in terms of reduction of platelet and white blood cell. Prior MTZ treatment reduces the risk of DCS to a level comparable to that we found in rats that did not ingested MNL. Several hypotheses may explain these observations.

Previous studies have shown that bubble formation and the incidence of DCS are highly dependent on body weight in rats^15^,^16^. Protocols using rats with a body weight above 350 g led to severe neurological symptoms and death. In our study, weights were similar in both groups and subgroups.

Dehydration is known to promote DCS^17^. Mannitol or 1.2.3.4.5.6-hexanehexol (C_6_H_14_O_6_) is a polyol (“sugar alcohol”). Following ingestion, it is not digested in the stomach or small intestine but is broken down in the large intestine through bacterial fermentation. We also know that 10% or 20% MNL solutions are hypertonic. High doses of MNL may have a laxative effect with subsequent fluid transfer through the gut wall^18^ leading to dehydration. Greater weight loss was not observed in rats force-fed with MNL compared to those force-fed with water. In addition, no significant differences were observed in hematocrit variation between rats force-fed with MNL and those force-fed with water. The MNL laxative effect was not sufficient to cause dehydration as a result of loss in feces.

The amount of hydrogen measured in exhaled air reflects the rate of colonic bacterial fermentation^14^. We have shown that ingesting MNL before diving significantly increases fermentation and exacerbates the risk of DCS. If MTZ is administered before force-feeding, fermentation is inhibited and the risk of DCS drops back down to baseline. To prove that the risk of DCS is related to bacterial fermentation, it has to be demonstrated that MTZ does not itself affect the risk of DCS. Several studies have shown that taking oral MTZ could have beneficial effects, not only local effects on inflammatory bowel disease^19^ but also systemic effects in subjects like burn victims^20^. This beneficial effect is not merely a result of MTZ’s antibiotic activity but also because of its antioxidant effect. This antioxidant effect may be related to modulation of the gut microbiota, specifically by stimulating growth of the population of bifidobacteria in the caecum^21^. In this study, no specific effect of MTZ was observed on the incidence of DCS so it seems that colonic bacterial fermentation during decompression after MNL ingestion promotes DCS. In addition, preliminary tests suggest that some non-fasting rats have a high spontaneous fermentation rate in the absence of MNL ingestion. Colonic bacterial fermentation could therefore be a general risk factor for DCS in rats, even those on a normal diet.

Hypercapnia (high partial carbon dioxide pressure [PaCO_2_] in the blood) is known to exacerbate the risk of DCS^22^. Colonic MNL fermentation is accompanied by the formation of carbon dioxide; some of this is eliminated via the anus but the rest crosses the intestinal barrier and diffuses throughout the body before being eliminated via the respiratory system. Before pressurization, there was no more CO_2_ in the air exhaled by rats force-fed with MNL than in that exhaled by those force-fed with water. In addition, there was no PaCO_2_ difference between rats force-fed with MNL and those force-fed with water. The higher incidence of DCS in rats force-fed with MNL cannot therefore be explained by hypercapnia.

A fraction of the hydrogen generated by MNL fermentation in the large intestine diffuses across the gut wall into the body and this endogenous hydrogen could act exacerbate the risk of DCS. It could directly increase the inert gas load during hyperbaric exposure and form bubbles during decompression. Before diving, it could also help to seed the initial formation of inert gas bubbles from nuclei—the micro-bubbles that are at the origin of desaturation events^2^.

Intraperitoneal administration of H_2_-rich saline 24 hours before a dive may have a protective effect against DCS in rats^23^. Recently, it was shown that successive deep dives impair endothelial function and enhance oxidative stress in humans^24^. Owing to its ability to rapidly diffuse across membranes, molecular hydrogen (H_2_) could reach and react with cytotoxic reactive oxygen species (ROS) and thus protect against oxidative damages. H_2_ has been recognized as a potential free radical scavenger which can selectively reduce the hydroxyl radical (OH∙) and peroxynitrite anion (ONOO-)^25^. Ohsawa *et al.* provided evidence that inhaled H_2_ had anti-oxidative and anti-apoptotic activities that protect the brain against ischemia-reperfusion injury and stroke^25^. Others have shown that H_2_ inhalation protects rabbits against spinal cord ischemia-reperfusion injury^26^. Thus, while increasing the inert gas load at the time of decompression appears to be deleterious, the place of endogenous hydrogen in the prevention and treatment of DCS remains to be explored.

## Conclusions

Colonic fermentation of MNL ingested before diving increases the incidence of DCS in fasting rats when the peak fermentation rate coincides with the decompression phase. More generally, colonic fermentation in rats receiving a normal diet without any MNL could be sufficient to increase the risk of DCS. The generation of hydrogen by fermentation and its subsequent diffusion through the body could increase this risk.

In humans, whether colonic fermentation during decompression promotes DCS remains to be assessed. Different foodstuffs are associated with differential fermentation rates so pre-dive dietary modification could cut down the risk of experiencing DCS. It is also known that the intestinal microbiota—including its fermentation capacity—can be modulated with the administration of pre- or pro-biotic agents. Finally, it might be worth investigating the effect of administering substances that absorb intestinal gases just before a dive.

## Materials and Methods

### Study population

All procedures involving experimental animals were in line with European Union rules (Directive 86/609) and French law (Decree 87/848). This study was approved by the IRBA Ethics Committee. The investigator (NV) has been accredited (Number 83.6) by the Health and Safety Directorate of our Department, as required by French rules R.214-93, R-214-99 and R.214-102.

Only male Sprague-Dawley rats (Charles River Laboratories, France) were used in this experiment in order to avoid fluctuations due to female hormone cycles. Rats were kept at 22 ± 1°C in a 12:00/12:00-h light/dark cycle (lights on at 7:00 a.m.) with food (Global Diet 2018, Harlan, Italy) and water *ad libitum*. Before experiments, rats were housed in an accredited animal care facility.

**Figure 4:**
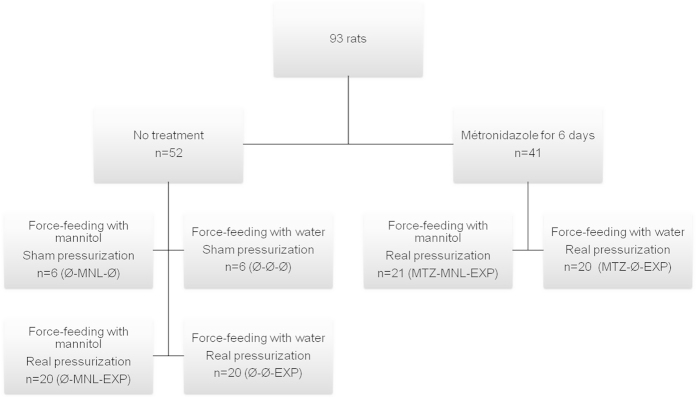
Distribution of rats in experimental groups and subgroups (MTZ: metronidazole, MNL: mannitol, EXP: exposed to pressurization).

**Figure 5:**
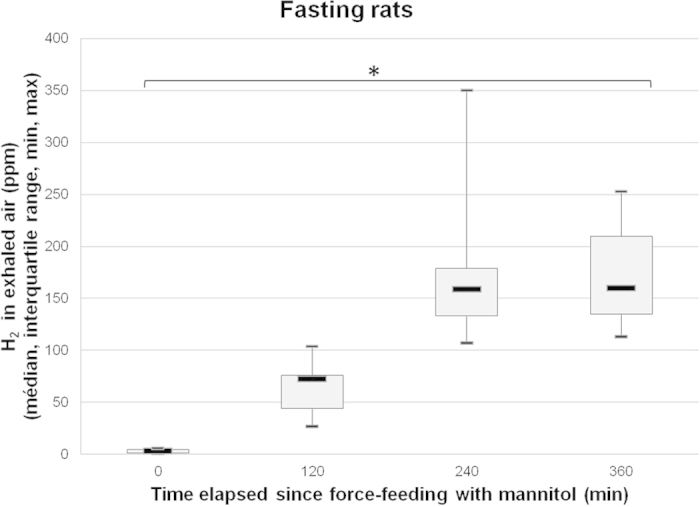
Distribution of rats in experimental groups and subgroups (MTZ: metronidazole, MNL: mannitol, EXP: exposed to pressurization).

A total of 93 rats were exposed to compressed air to induce decompression stress and bubble formation. The rats were randomly divided into two groups, the first one (n = 41) was treated for six days before pressurization with MTZ, administered as a solution contained 250 mg MTZ and 6 g sucrose per 100 ml; the expected daily MTZ intake was 40 mg per rat or 100 mg.kg^−1^. This dose is higher than the dose providing a significant decrease in pulmonary hydrogen excretion after lactulose ingestion in humans and animals^27^,^28^,^29^. The second group (n = 52) was given a control solution containing 6 g sucrose per 100 ml. Twelve animals in this second group were subjected to sham pressurization (with no pressure increase in the chamber). Each group was further randomly divided into an experimental subgroup and a control subgroup (Fig. 4). Four hours before pressurization, rats in the experimental subgroup were force-fed with a bolus of 2.5 ml of 20% MNL solution (0.5 g or 0.125 g.100 g^−1^) while the control subgroup was force-fed with 2.5 ml of water, so that decompression coincided with the peak of hydrogen exhalation. Indeed we determined in a preliminary test that exhaled hydrogen peaked 4–6 hours after MNL ingestion (Fig. 5), while the peak was observed 8 hours after lactulose ingestion in gnotobiotic rats^30^. The dose was also determined from preliminary tests in which a rise in exhaled hydrogen was observed quite similar – but with less inter individual variability – to the one obtained in non-fasting rats force-fed with 2.5 ml of water (135 ± 47 ppm versus 76 ± 126 ppm; NS) (see supplementary information).

### Hyperbaric procedure

Batches of 8 freely-moving rats in two different cages (4 per cage) were subjected to a hyperbaric protocol in a 200-liter hyperbaric chamber fitted with three ports for observation. Each batch consisted of a heterogeneous sample of rats which had been treated with MTZ or not and had been pre-fed with either MNL or water.

We used a staged decompression, capable of causing ischemic impairments of the spinal cord with variable severity in the rat^31^,^32^. This model therefore makes it possible to move closer to the DCS observed in humans. In fact, in recreational diving medullary DCS is frequent and, in most cases, occurs in the absence of a fault in procedure or a fast ascent to the surface^8^.

Compression was induced at a rate of 100 kPa.min^−1^ to a pressure of 1000 kPa (90 msw) and then maintained for 45 minutes in air. At the end of the exposure period, the rats were decompressed down to 200 kPa at a rate of 100 kPa.min^−1^ with a 5-minute stop at 200 kPa, a 5-minute stop at 160 kPa, and a 10-minute stop at 130 kPa. Decompression between 200 kPa and surface was performed at a rate of 10 kPa.min^−1^. The decompression rate was automatically controlled by a computer linked to an Analogical/Digital converter (NIUSB-6211^®^, National Instrument, USA) itself connected to a solenoid valve (Belino LR24A-SR^®^, Switzerland) and a pressure transmitter (Pressure Transmitter 8314, Burket Fluid Control System, Germany). The program used to control the decompression rate was designed in-house on DasyLab (DasyLab National Instrument, USA).

Compressed air was generated using a diving compressor (Mini Verticus III, Bauer Comp, Germany) coupled to a 100-liter hyperbaric chamber at 3.10^4^ kPa. The oxygen analyzer was based on a MicroFuel electrochemical cell (G18007 Teledyne Electronic Technologies/Analytical Instruments, USA). Water vapor and CO_2_ produced by the animals were respectively captured with Seccagel (relative humidity: 40–60%) and soda lime (<300 ppm captured by the soda lime), respectively. Gases were mixed by an electric fan. The day-night cycle was respected throughout. The temperature inside the hyperbaric chamber was measured using a platinum-resistance temperature probe (Pt 100, Eurotherm, France). All these variables were controlled by a dedicated computer.

### Hydrogen and carbon dioxide in exhaled air

The hydrogen in the exhaled air derives from the fermentation of carbohydrate in the colon. The carbon dioxide derives from metabolic processes as well as fermentation processes in the gut.

To measure hydrogen and carbon dioxide in exhaled air, we assumed that the ventilation rate was constant over time and the same in all rats, i.e. 225 ml.min^−1^: a resting value of 27.27 ± 2.39 ml.min^−1^.100g^−1^ (mean standard deviation) has been documented for Sprague-Dawley rats^33^.

For each measurement, rats were individually introduced at surface pressure into a clean, dry polyvinyl chloride (PVC) cylinder (internal diameter 75 mm, length 200 mm, internal volume 883 mL). Both sides were sealed by plastic disks with holes. Air was insufflated at one side and collected at the other one. Air was supplied by an aerator (Rena Air 200^®^, France) at a constant rate of 225 ml.min^−1^ (checked by the rise of a bubble in an inverted 100 ml test-tube, pierced at the bottom).

After 5 min to allow the mixing of the gases inside the cylinder (taking into account the dead space not occupied by the rat), hydrogen (ppm) and carbon dioxide (percentage) were measured in the air coming out of the cylinder.

Hydrogen was measured using a portable expired hydrogen analyzer (Gastrolyser^®^, Bedfont Scientific Ltd, UK). Carbon dioxide was measured using a portable gas exchange analysis system (Cosmed^®^ K4b^2^, Italy). Data were recorded four hours before compression just before force-feeding and one hour before compression.

### Behavior and clinical observations

At the end of the decompression, each rat was put into an individual cage and assessed at will for 30 minutes by observers who were unaware of the animal’s pre-compression conditioning. The following symptoms were considered as manifestations of DCS: labored breathing, paresis or difficulty moving (including limping, failure to maintain balance, sideways gait, falling, difficulty getting up after a fall) or death. Rats were considered as having a paresis when they didn’t reach grade 4 of the “Motor Score” presented by Gale *et al.*^34^. Times of onset were also recorded. Problems with fore or rear limbs were classified as being due to neurological DCS.

### Blood cell counts

Blood cell counts were carried out in an automatic analyzer (ABCvet^®^, SCIL, France) on samples taken 4 hours before the dive and then again 30 minutes after surfacing. Red cells, leukocytes and platelets were counted in 20 μl samples taken from the tip of the tail and diluted in an equivalent volume of 2 mM EDTA (Sigma, France).

### Arterial blood gases

The rats were then anesthetized by an intraperitoneal injection of a mixture of 10 mg.kg^−1^ xylazine (Rompum^®^ 2%, Bayer Pharma, Germany) and 100 mg.kg^−1^ ketamine (Imalgene^®^1000, Rhône laboratory, France).

A sample of arterial blood was taken from each rat by direct aortic puncture and blood gases were measured using an i-STAT System (Abbott Laboratories, Illinois, USA).

The rats were sacrificed by injecting pentobarbital (200 mg.kg^−1^ ip, Sanofi Santé, France).

### Statistical analyses

Statistical processing was done with XLSTAT-Pro^®^ (Addinsoft, Paris, France). Numerical results were expressed as median interquartile range for quantitative variables, and percentage confidence interval [95%] for dichotomous variables. A contingency table was used for independence and association tests, coupled with a Fisher Exact or Chi^2^ test of significance. Differences between two groups were analyzed using a Mann-Whitney test and a Wilcoxon test was used for matched comparisons within groups. A difference was considered as significant for p-values < 0.05.

## Additional Information

**How to cite this article**: de Maistre, S. *et al.* Colonic Fermentation Promotes Decompression Sickness in Rats. *Sci. Rep.* **6**, 20379; doi: 10.1038/srep20379 (2016).

## Supplementary Material

Supplementary Information
