# Supplementary material for: Colonic Fermentation Promotes Decompression sickness in Rats
Source: Sci Rep. 2016 Feb 8;6:20379. doi: 10.1038/srep20379 (PMC4745042; doi:10.1038/srep20379)
Supplement: Supplementary Information [file srep20379-s1.pdf]

COLONIC FERMENTATION PROMOTES DECOMPRESSION SICKNESS IN RATS

Authors : de MAISTRE[1*] Sébastien, VALLEE[2] Nicolas, GEMPP[1] Emmanuel, LAMBRECHTS[2] Kate, LOUGE[1] Pierre, DUCHAMP[3] Claude, BLATTEAU[2] Jean-Eric.

Affiliations :

[1] Service de Médecine Hyperbare et Expertise Plongée, HIA Sainte-Anne, BP600, Toulon Cedex 9, France

[2] Équipe Résidente de Recherche Subaquatique Opérationnelle, Institut de Recherche Biomédicale des Armées, BP 600, Toulon Cedex 9, France

[3] LEHNA, UMR 5023-CNRS/UCBL, Université Claude Bernard Lyon 1, 43 Bd du 11 Novembre 1918, Villeurbanne Cedex, France

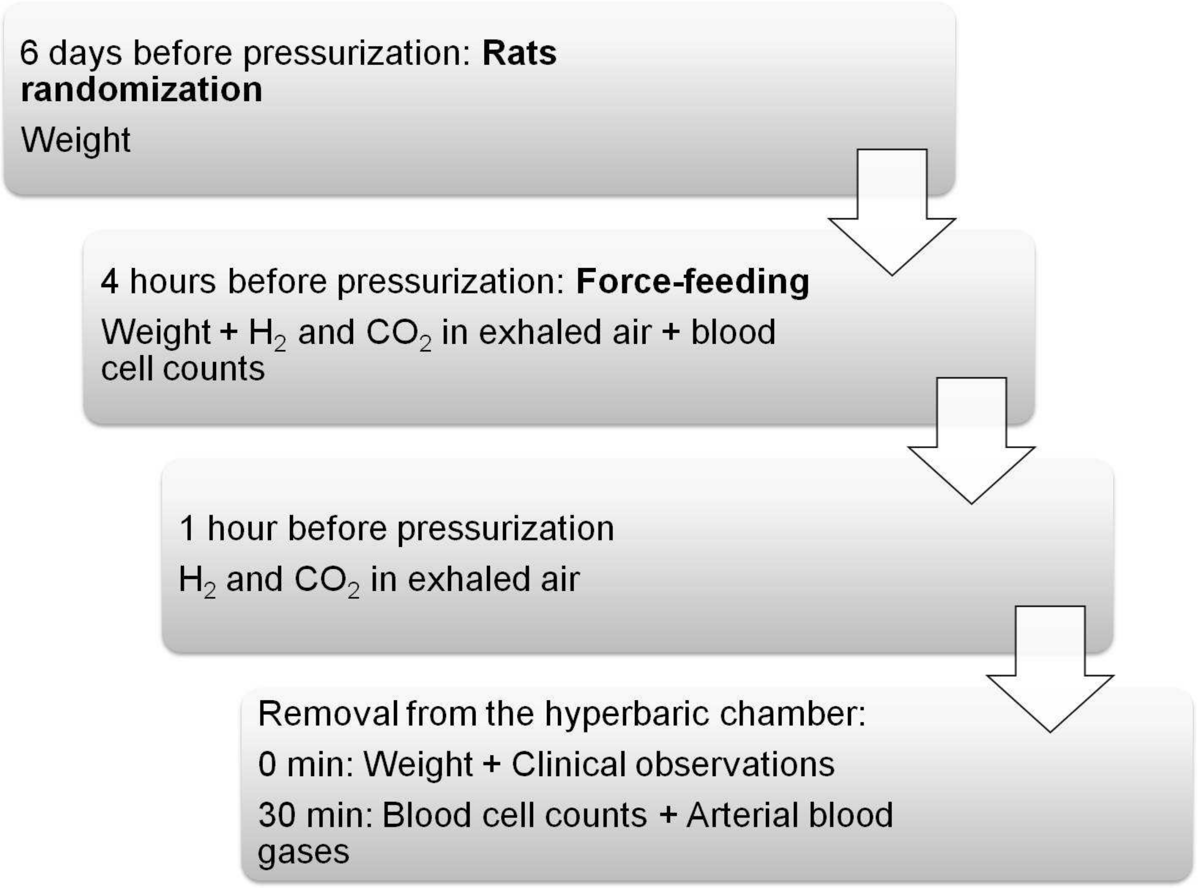

Figure 1: Flow chart describing the experimental design

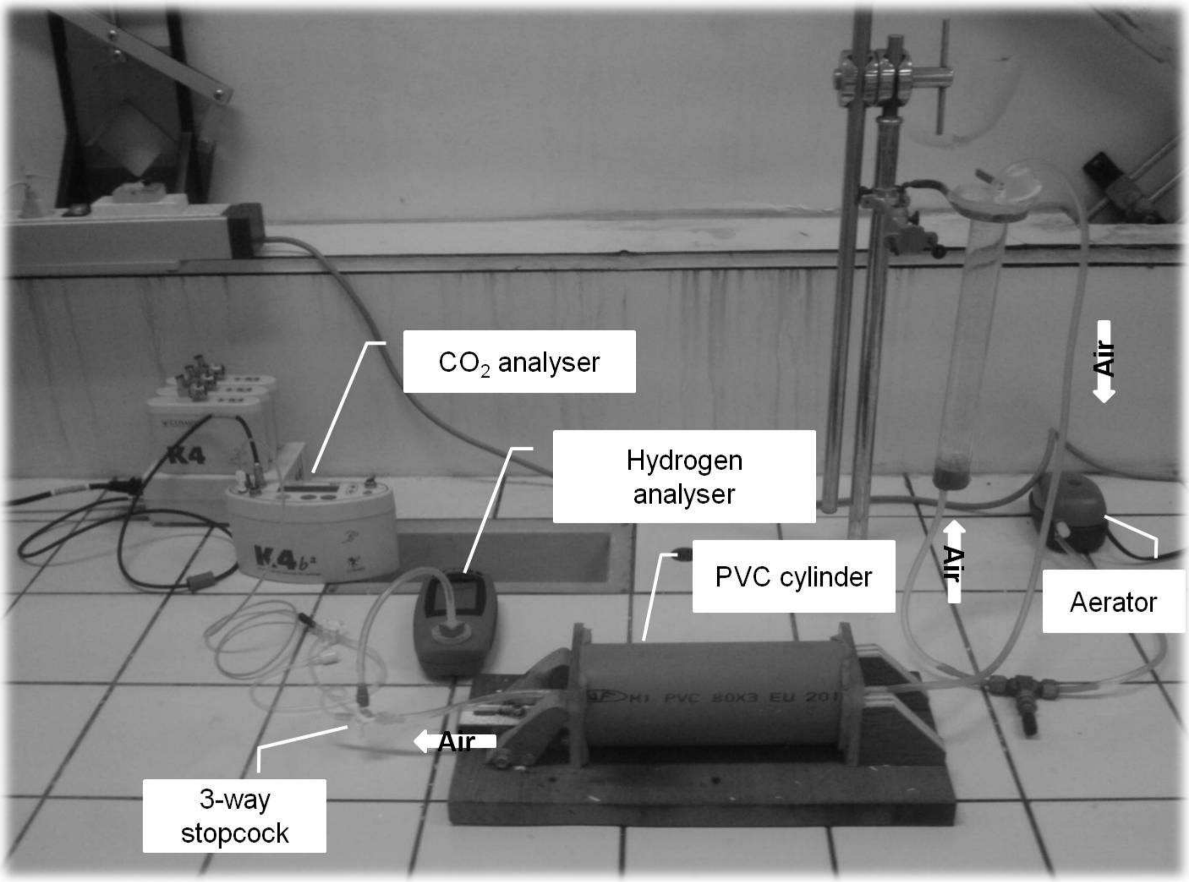

Figure 2: Installation used for the measurement of hydrogen and carbon dioxide in exhaled air in rats.

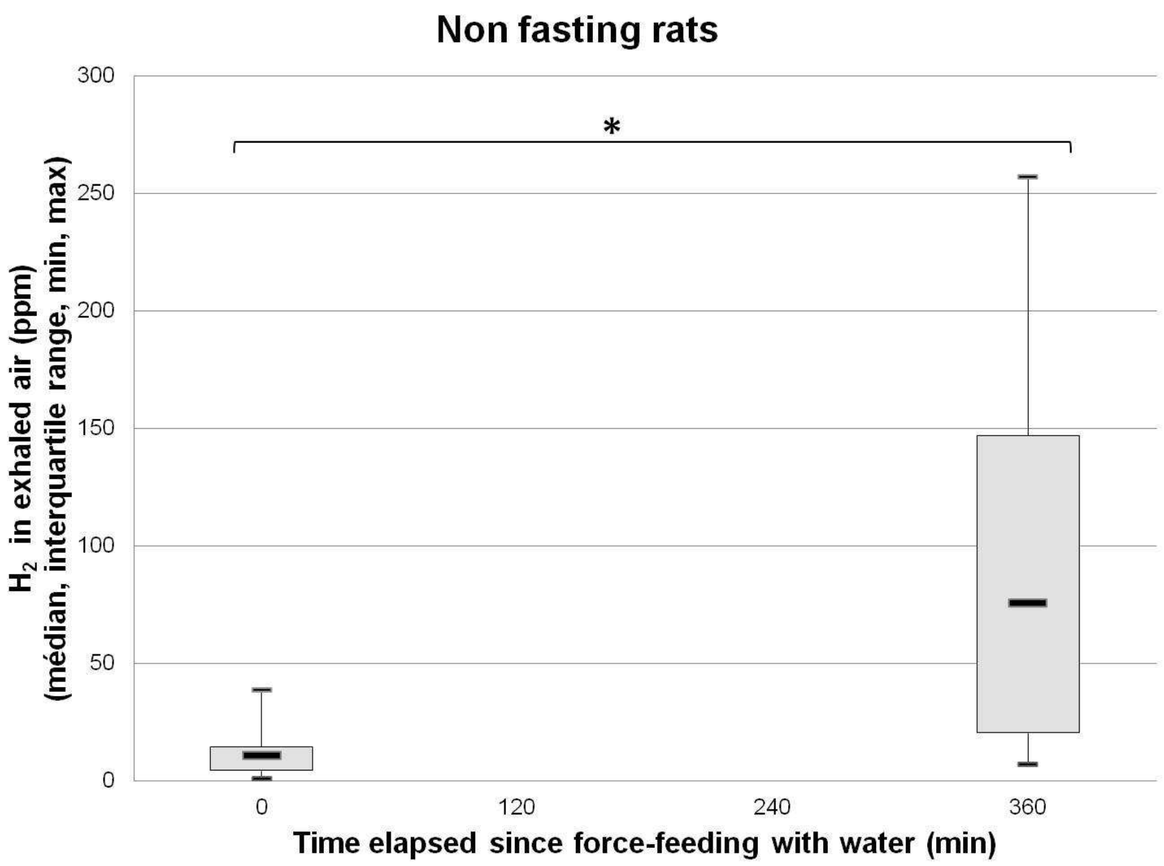

Figure 3 : Hydrogen in exhaled air after force-feeding with 2.5 ml of water in non fasting rats (n=12). *denotes p<0.05 between the paired rats.
